# Selective Oxidation of Methane to Methanol via In Situ H_2_O_2_ Synthesis

**DOI:** 10.1021/acsorginorgau.3c00001

**Published:** 2023-04-20

**Authors:** Fenglou Ni, Thomas Richards, Louise R. Smith, David J. Morgan, Thomas E. Davies, Richard J. Lewis, Graham J. Hutchings

**Affiliations:** † Max Planck−Cardiff Centre on the Fundamentals of Heterogeneous Catalysis FUNCAT, Cardiff Catalysis Institute, School of Chemistry, Cardiff University, Main Building, Park Place, Cardiff CF10 3AT, United Kingdom; ‡ Research Complex at Harwell (RCaH), Harwell XPS, Didcot OX11 0FA, U.K.

**Keywords:** methane oxidation, hydrogen peroxide, green chemistry, gold, palladium

## Abstract

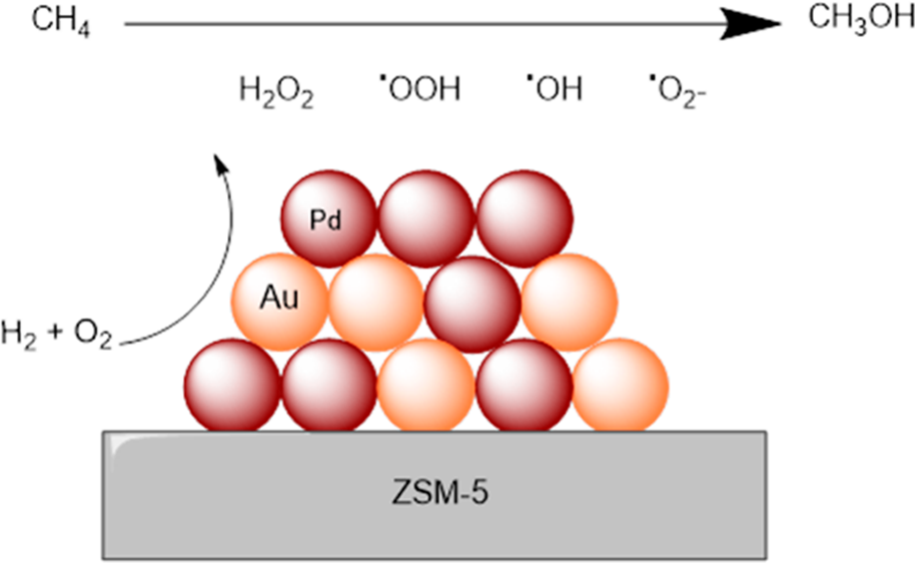

The selective oxidation of methane to methanol, using H_2_O_2_ generated in situ from the elements, has been investigated using a series of ZSM-5-supported AuPd catalysts of varying elemental composition, prepared via a deposition precipitation protocol. The alloying of Pd with Au was found to offer significantly improved efficacy, compared to that observed over monometallic analogues. Complementary studies into catalytic performance toward the direct synthesis and subsequent degradation of H_2_O_2_, under idealized conditions, indicate that methane oxidation efficacy is not directly related to H_2_O_2_ production rates, and it is considered that the known ability of Au to promote the release of reactive oxygen species is the underlying cause for the improved performance of the bimetallic catalysts.

## Introduction

Natural gas has long been considered a bridging feedstock to enable a transition away from a petroleum-dependent global economy. However, the selective valorization of its principal components (methane and ethane), under reaction conditions that are environmentally benign, is yet to be realized. In particular, the oxidation of methane to methanol, a valuable platform chemical with global demand estimated at 22 billion gallons/annum,^1^ represents a long-standing challenge of catalysis, which is perhaps more pertinent now more than ever given aspirations to reach net zero carbon emissions. Indeed, with 142 BCM of natural gas flared in 2020 (equivalent to approximately 4% of global production^2^), the selective oxidation of methane is set to continue to remain a major area of research interest for the foreseeable future.

Currently, industrial methanol production is dominated by an energy-intensive two-step process, where methane is first converted to syngas (CO and H_2_). In recent years the valorisation of methane via the in situ production of H_2_O_2_ has been an area of considerable academic interest,^3−6^ utilizing lower temperatures (<80 °C) to alternative thermal catalytic routes.^7−10^ In particular, the in situ approach would offer improved economic viability compared to the use of the preformed oxidant, with the cost of commercial H_2_O_2_ typically being in excess of methanol itself. Additionally, the in situ route has been widely reported to offer improved methanol selectivity compared to that observed when using ex situ H_2_O_2_,^11^ although in general methanol formation rates are often considerably lower when H_2_O_2_ is generated in situ from the elements.^5^

The use of the aluminosilicate ZSM-5 within methane oxidation has received considerable attention, while such studies have typically focused on biomimetic oxidation, utilizing Fe and/or Cu species incorporated into the zeolite framework in conjunction with preformed H_2_O_2_.^12−17^ Additionally, there has been growing attention placed on the use of ZSM-5 as a support for active metal species for both the direct synthesis of H_2_O_2_^18−22^ and in situ oxidation of methane to methanol.^23^ Recently Jin et al. reported that enhanced rates of methane oxidation via in situ H_2_O_2_ synthesis can be achieved through the introduction of a hydrophobic organo-silane layer onto the external surface of a AuPd@ZSM-5 catalyst, with the improved reactivity attributed to the increased localized concentrations of reagents (H_2_ and O_2_), and the confinement of the subsequently synthesized H_2_O_2_ and CH_4_ in close proximity to active sites.^24^

With these earlier studies in mind, we now investigate the activity of AuPd nanoalloys immobilized onto a commercially available ZSM-5 support for the valorization of methane via the in situ production of H_2_O_2_, with an aim to gain further insight into the efficacy of such catalytic systems and further develop an in situ approach to alkane upgrading.

## Results and Discussion

Our initial studies via X-ray diffraction (XRD) (Figure S1, supplementary note 1) and Fourier transform infrared (FTIR) spectroscopy (Figure S2, supplementary note 2) established that the synthesis and thermal treatment of the AuPd/ZSM-5 catalysts resulted in no significant detrimental effects on zeolitic structure, as evidenced by comparison to the as-supplied ZSM-5 support. Notably, our XRD analysis revealed no clear reflections associated with immobilized metals, which may be expected given the low total metal loading of these materials. The textural properties of key synthesized catalysts are summarized in Table S1. In keeping with previous investigations^25^ the immobilization of active metals resulted in a general decrease in both total surface area and micropore volume in comparison to the bare zeolitic material, with this attributed to the deposition of metal species within the zeolitic pore structure.

Using reaction conditions that have previously been optimized to promote H_2_O_2_ stability,^26^ our initial studies established the effect of Au/Pd ratio (actual metal loading, as determined by digestion of as-prepared catalysts and MP-AES analysis reported in Table S2) on catalytic reactivity toward the direct synthesis and subsequent degradation of H_2_O_2_ (Table 1, an investigation into catalyst reusability is presented in Table S3). In keeping with recent works into AuPd nanoalloys supported on zeolite^25^ and silica supports,^27^ we observed a direct correlation between catalytic activity toward both the direct synthesis and subsequent degradation of H_2_O_2_ and Pd content, with the 0.5%Pd/ZSM-5 catalyst offering rates of H_2_O_2_ production (12 mol_H_2_O_2__ kg_cat_^–1^ h^–1^) somewhat higher than 0.25%Au-0.25%Pd/ZSM-5 analogue (9 mol_H_2_O_2__ kg_cat_^–1^ h^–1^). However, the bimetallic catalyst was found to be far more selective, with H_2_O_2_ degradation rates (68 mol_H_2_O_2__ kg_cat_^–1^ h^–1^) significantly lower than those of the Pd-only catalyst (112 mol_H_2_O_2__ kg_cat_^–1^ h^–1^).

**Table 1:** Catalytic Activity of 0.5%AuPd/ZSM-5 Catalysts toward the Direct Synthesis and Subsequent Degradation of H_2_O_2_ as a Function of the Au/Pd Ratio^a^

catalyst	productivity (mol_H_2_O_2__ kg_cat_^–1^ h^–1^)	H_2_O_2_ conc. (ppm)	degradation (mol_H_2_O_2__ kg_cat_^–1^ h^–1^)
ZSM-5	0	0	12
0.5%Au/ZSM-5	2	100	20
0.475%Au-0.025%Pd/ZSM-5	3	110	35
0.375%Au-0.125%Pd/ZSM-5	7	460	58
0.25%Au-0.25%Pd/ZSM-5	9	720	68
0.125%Au-0.375%Pd/ZSM-5	10	732	104
0.025%Au-0.475%Pd/ZSM-5	12	761	112
0.5%Pd/ZSM-5	12	786	116

a H_2_O_2_ direct synthesis reaction conditions: catalyst (0.01 g), H_2_O (2.9 g), MeOH (5.6 g), 5% H_2_/CO_2_ (420 psi), 25% O_2_/CO_2_ (160 psi), 0.5 h, 2 °C, 1200 rpm. H_2_O_2_ degradation reaction conditions: catalyst (0.01 g), H_2_O_2_ (50 wt %, 0.68 g), H_2_O (2.22 g), MeOH (5.6 g), 5% H_2_/CO_2_ (420 psi), 0.5 h, 2 °C, 1200 rpm.

**Figure 1:**
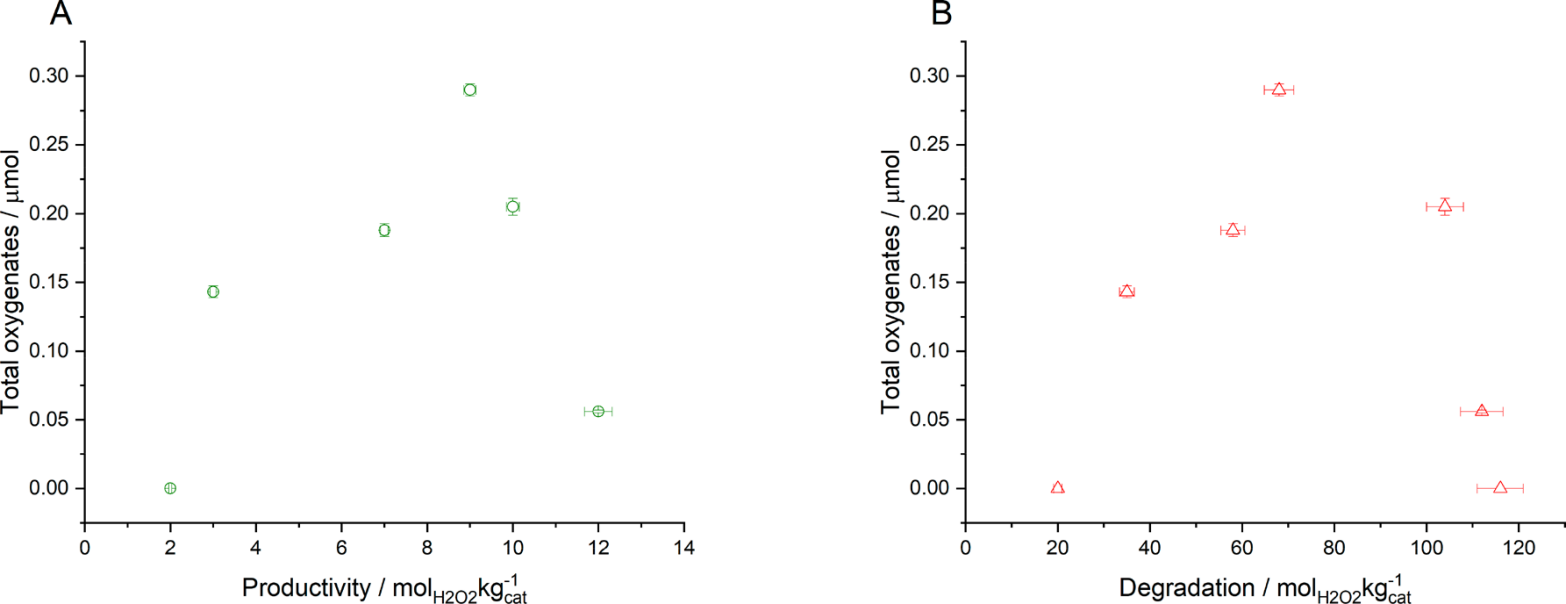
Correlation between catalytic activity toward the in situ selective oxidation of methane and (A) the direct synthesis and (B) subsequent degradation of H_2_O_2_. H_2_O_2_ direct synthesis reaction conditions: catalyst (0.01 g), H_2_O (2.9 g), MeOH (5.6 g), 5% H_2_/CO_2_ (420 psi), 25% O_2_/CO_2_ (160 psi), 0.5 h, 2 °C, 1200 rpm. H_2_O_2_ degradation reaction conditions: catalyst (0.01 g), H_2_O_2_ (50 wt %, 0.68 g), H_2_O (2.22 g), MeOH (5.6 g), 5% H_2_/CO_2_ (420 psi), 0.5 h, 2 °C, 1200 rpm. Methane oxidation reaction conditions: catalyst (0.027 g), H_2_O (10.0 g), 435 psi total pressure (0.8% H_2_/1.6% O_2_/76.7% CH_4_/20.8% N_2_), 0.5 h, 50 °C, 1500 rpm.

Evaluation of the catalytic series toward the oxidation of methane, via in situ H_2_O_2_ production, is reported in Table 2, with a comparison of catalytic performance toward oxygenate formation during the in situ oxidation of methane and activity toward H_2_O_2_ synthesis and degradation reported in Figure 1A,B. A wider comparison of the catalytic performance of the materials investigated in this work to those previously investigated in the literature using in situ-generated H_2_O_2_ is reported in Table S.4. Catalytic activity toward methane oxidation was not found to follow the same trend as that observed for H_2_O_2_ direct synthesis (Table 1), with the bimetallic formulations achieving higher rates of methane oxidation than that observed over the monometallic Pd catalyst, despite the greater activity of this formulation toward H_2_O_2_ synthesis. Indeed, it is notable that the Pd-only catalyst displayed no activity toward the in situ oxidation of methane. Such observations may indicate that the observed reactivity trends for in situ methane oxidation may not be wholly related to H_2_O_2_ production. When considered alongside earlier works which have reported the ability of Au to promote the desorption of reactive oxygen species (•OOH, •OH, and •O_2_^–^),^28,29^ in addition to H_2_O_2_,^30^ from catalytic surfaces. It is possible to suggest that in addition to H_2_O_2_, there is a significant contribution to the observed catalysis from intermediate species generated during the formation of H_2_O_2_. Such observations would align well with earlier works that have identified the crucial role of •OH formation, from H_2_O_2_, and the resulting activation of methane via H-abstraction.^31^ It should be noted that regardless of catalyst formulation, total selectivity toward methanol was observed, which can be related to the relatively low reactivity of the materials studied.

**Table 2:** Effect of Au/Pd Ratio on the Activity of 0.5%AuPd/ZSM-5 Catalysts toward the Oxidation of Methane to Methanol via In Situ H_2_O_2_ Production^a^

	products (μmol)					
catalyst	CH_3_OH	CH_3_OOH	HCOOH	CO_2_	productivity (μmol_oxygenates_ g_cat_^–1^)	TOF (h^–1^)
ZSM-5	0	0	0		0	0
0.5%Au/ZSM-5	0	0	0		0	0
0.475%Au-0.025%Pd/ZSM-5	0.143	0	0		5.3	0.39
0.375%Au-0.125%Pd/ZSM-5	0.188	0	0		7.0	0.44
0.25%Au-0.25%Pd/ZSM-5	0.290	0	0		10.7	0.57
0.125%Au-0.375%Pd/ZSM-5	0.205	0	0		7.6	0.35
0.025%Au-0.475%Pd/ZSM-5	0.056	0	0		2.1	0.09
0.5%Pd/ZSM-5	0	0	0		0	0

a Methane oxidation reaction conditions: catalyst (0.028 g), H_2_O (10.0 g), 435 psi total pressure (0.8% H_2_/1.6% O_2_/76.7% CH_4_/20.8% N_2_), 0.5 h, 50 °C, 1500 rpm. Note 1: For all catalyst formulations, CO_2_ production was found to be within experimental error of the blank reaction. Note 2: Turnover frequency (TOF) calculated using the total moles of product and based on theoretical metal loading.

The selectivity of Pd-based catalysts toward H_2_O_2_ has been widely reported to be related to particle size, with the high proportion of defect sites present in smaller particles considered to be responsible for H_2_O_2_ degradation.^32,33^ Similar activity trends have also been reported for the selective oxidation of methane to methanol when supported AuPd nanoalloys were utilized in conjunction with preformed H_2_O_2_.^3^ Determination of the mean nanoparticle size of key catalysts via transmission electron microscopy (TEM) (Table 3, with corresponding electron micrographs reported in Figure S3) reveals a minimal variation of this metric across the catalytic series. As such it is possible to conclude that catalytic trends are not dependent on particle size effects.

**Table 3:** Mean Particle Size of 0.5%AuPd/ZSM-5 Catalysts as Determined via TEM^a^

catalyst	mean particle size (nm) (standard deviation)
0.5%Au/ZSM-5	6.5 (2.2)
0.25%Au-0.25%Pd/ZSM-5	4.1 (1.3)
0.5%Pd/ZSM-5	4.1 (1.1)

a Note: All catalysts were exposed to a reductive heat treatment (3 h, 400 °C, 10 °C min^–1^, 5%H_2_/Ar).

X-ray photoelectron spectroscopic (XPS) evaluation of the as-prepared AuPd/ZSM-5 catalysts and analogous spent materials is shown in Table 4, with representative spectra reported in Figure S4. Evident from our analysis is the marked difference in the Au/Pd ratio between the fresh and used catalysts, although in all cases both Au and more interestingly Pd are found to exist in the metallic state, with the enhanced activity of Pd^0^ species, compared to Pd^2+^ analogues well reported for H_2_O_2_ direct synthesis.^34^ Subtle changes in the Si/Al ratio should also be noted and are indicative of an increase in the surface Al content. In all instances, after use in the methane oxidation reaction, the amount of Au was observed to decrease relative to that of Pd, with such observations possibly indicative of the agglomeration or leaching of metal species.

**Table 4:** XPS-Derived Au/Pd and Si/Al Ratios for Fresh and Used 0.5%AuPd/ZSM-5 Catalysts^a^

	Au/Pd		Si/Al	
catalyst	fresh	used	fresh	used
0.5%Au/ZSM-5	n/a	n/a	11.4	12.4
0.475%Au-0.025%Pd/ZSM-5	13.0	n/d	11.4	12.4
0.375%Au-0.125%Pd/ZSM-5	0.78	0.25	12.1	12.5
0.25%Au-0.25%Pd/ZSM-5	0.50	0.07	12.1	12.6
0.125%Au-0.375%Pd/ZSM-5	0.17	0.36	11.9	12.3
0.025%Au-0.475%Pd/ZSM-5	n/d	n/d	11.2	11.8
0.5%Pd/ZSM-5	n/a	n/a	11.7	12.4

a Note: n/a = not applicable, n/d = unable to determine due to low concentration of metal.

**Figure 2:**
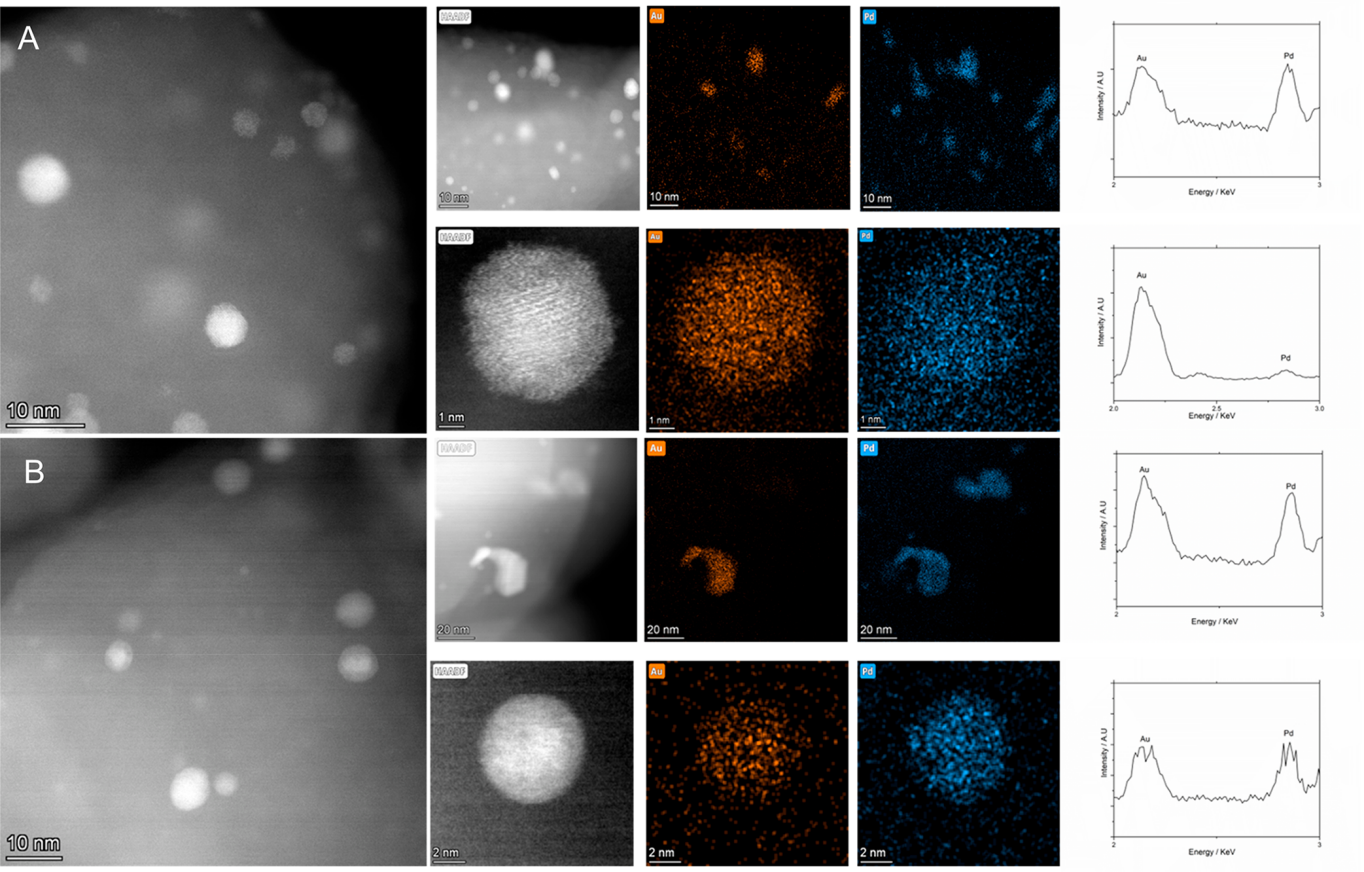
High-angle annular dark-field scanning transmission electron microscopy analysis of the (A) as-prepared 0.25%Au-0.25%Pd/ZSM-5 catalyst and (B) after use in the in situ oxidation of the methane reaction. Corresponding EDX mapping and spectra are also reported [Au (Mα) and Pd (Lα)] centered at 2.12 and 2.84 keV, respectively. The larger particles are shown to be Au-rich AuPd alloys and the smaller particles primarily Pd.

Given the potential for structural modification or metal leaching indicated by our analysis by XPS, we subsequently conducted a detailed analysis of the as-prepared 0.25%Au-0.25%Pd/ZSM-5 catalyst after use in the in situ methane oxidation reaction (Figure 2A,B). Such analysis identified a broad particle size distribution with the larger particles (>5 nm) observed to be Au-rich AuPd alloys, primarily of a Au-core, Pd-shell morphology. By comparison, the smaller nanoparticles (<5 nm) were found to consist of Pd only. Notably, no clear variation in particle composition or size was observed between the fresh and used samples. We subsequently investigated the stability of the catalytic series through MP-AES analysis of post-reaction solutions (Table 5). These studies confirmed the loss of both Au and Pd upon use, which may explain the elemental variation observed via XPS analysis; however, the extent of such leaching was relatively low. Regardless, it is clear that further efforts to promote catalyst stability are still required, in particular given the known activity of homogeneous metal species to catalyze both the direct synthesis of H_2_O_2_^35^ and the oxidation of methane.^36^

**Table 5:** Metal Leaching during the Selective Oxidation of Methane via In Situ H_2_O_2_ Synthesis, as Determined from MP-AES Analysis of Postreaction Solutions^a^

	metal leached (%)	
catalyst	Au	Pd
0.5%Au/ZSM-5	1.4	
0.475%Au-0.025%Pd/ZSM-5	0.9	2.1
0.375%Au-0.125%Pd/ZSM-5	0.6	1.1
0.25%Au-0.25%Pd/ZSM-5	0.4	0.6
0.125%Au-0.375%Pd/ZSM-5	3.6	4.2
0.025%Au-0.475%Pd/ZSM-5	1.8	1.8
0.5%Pd/ZSM-5		1.5

a Methane oxidation reaction conditions: catalyst (0.027 g), H_2_O (10.0 g), 435 psi total pressure (0.8% H_2_/1.6% O_2_/76.7% CH_4_/20.8% N_2_), 0.5 h, 50 °C, 1500 rpm.

## Conclusion

The selective valorization of methane to methanol via the in situ synthesis of H_2_O_2_ represents an attractive, low-temperature alternative to current industrial routes to this platform chemical and would avoid the significant costs associated with the use of commercial H_2_O_2_. Within this work, using low-loaded AuPd nanoalloys immobilized onto a ZSM-5 support, we demonstrate the key role of Au and Pd content on catalytic performance, with materials which consist of a Au/Pd ratio approaching 1:1 shown to offer enhanced reactivity. This is despite the improved performance of the Pd-only analogue toward H_2_O_2_ production, under conditions idealized for H_2_O_2_ stability. Such trends may indicate the key role of alternative reactive oxygen species for methane oxidation and would align well with recent findings. While catalyst stability is of concern, we consider that these materials represent a promising basis for further exploration for the selective oxidation of a range of feedstocks, in particular given their high selectivity toward methanol.

## Experimental Section

### Catalyst Preparation

Prior to co-deposition of metal salts, NH_4_-ZSM-5 (Zeolyst) was calcined in flowing air (450 °C, 6 h, 3 °C min^–1^) according to our previous work.^37^ Mono- and bimetallic 0.5%Au–Pd/ZSM-5 catalysts have been prepared (on a weight metal basis) by the co-deposition of metal salts, based on a methodology previously reported in the literature.^7^ The procedure to produce the 0.25%Au–0.25%Pd/ZSM-5 catalyst (1 g) is outlined below.

PdCl_2_ (0.42 mL, [Pd] = 6 mg mL^–1^, Sigma-Aldrich) and HAuCl_4_·3H_2_O solution (0.21 mL, [Au] = 12.25 mg mL^–1^, Strem Chemicals) were charged into deionized water (67 mL), under stirring (600 rpm), followed by the addition of the ZSM-5 support (0.995 g). Subsequently, NH_4_OH (2.5 wt %) was added dropwise over 30 min, finally reaching a pH of 6. The temperature of the resulting slurry was increased to 60 °C and aged for 2 h with stirring (600 rpm), followed by separation of the solid catalyst via filtration and subsequent washing with deionized water (800 mL). The recovered catalyst was then ground and dried under vacuum (60 °C, 16 h) prior to heat treatment (5%H_2_/Ar, 400 °C, 3 h, 10 °C min^–1^).

### Catalyst Testing

#### Note 1:

For both H_2_O_2_ direct synthesis and degradation experiments, the reactor temperature was controlled using a HAAKE K50 bath/circulator using an appropriate coolant. Reactor temperature was maintained at 2 ± 0.2 °C throughout the course of the H_2_O_2_ synthesis and degradation reaction.

#### Note 2:

The conditions used within this work for H_2_O_2_ synthesis and degradation have previously been investigated, with the use of subambient reaction temperatures, CO_2_ reactant gas diluent and a methanol co-solvent identified as key to maintaining high catalytic efficacy toward H_2_O_2_ production.^26^ In particular the CO_2_ gaseous diluent, has been found to act as an in situ promoter of H_2_O_2_ stability through dissolution in the reaction solution and the formation of carbonic acid. We have previously reported that the use of the CO_2_ diluent has a comparable promotive effect to that observed when acidifying the reaction solution to a pH of 4 using HNO_3_.^38^

#### Note 3:

In all cases, reactions were run multiple times, over multiple batches of catalyst, with the data being presented as an average of these experiments.

### Direct Synthesis of H_2_O_2_

Hydrogen peroxide synthesis was evaluated by using a Parr Instruments stainless steel autoclave with a nominal volume of 100 mL and a maximum working pressure of 2000 psi. To test each catalyst for H_2_O_2_ synthesis, the autoclave was charged with catalyst (0.01 g) and solvent (5.6 g methanol and 2.9 g H_2_O, Fischer Scientific, HPLC standard). The charged autoclave was then purged three times with 5% H_2_/CO_2_ (100 psi) before filling with 5% H_2_/CO_2_ to a pressure of 420 psi, followed by the addition of 25% O_2_/CO_2_ (160 psi). The reaction was conducted at a temperature of 2 °C, for 0.5 h with stirring (1200 rpm). Reactant gases were not continuously supplied. H_2_O_2_ productivity was determined by titrating aliquots of the final solution after reaction with acidified Ce(SO_4_)_2_ (0.0085 M) in the presence of a ferroin indicator. Catalyst productivities are reported as mol_H_2_O_2__ kg_cat_^–1^ h^–1^.

Catalytic conversion of H_2_ and selectivity toward H_2_O_2_ were determined using a Varian 3800 GC fitted with TCD and equipped with a Porapak Q column.

H_2_O_2_ selectivity (eq 1) is defined as follows:
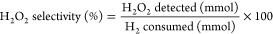
1

### Degradation of H_2_O_2_

Catalytic activity toward H_2_O_2_ degradation was determined in a similar manner to the direct synthesis activity of a catalyst. The autoclave was charged with methanol (5.6 g, Fischer Scientific, HPLC standard), H_2_O_2_ (50 wt % 0.68 g, Merck), H_2_O (2.22 g, Fischer Scientific HPLC standard), and catalyst (0.01 g), with the solvent composition equivalent to a 4 wt % H_2_O_2_ solution. From the solution, two aliquots of 0.05 g were removed and titrated with acidified Ce(SO_4_)_2_ solution using ferroin as an indicator to determine an accurate concentration of H_2_O_2_ at the start of the reaction. The autoclave was pressurized with 5% H_2_/CO_2_ (420 psi). The reaction was conducted at a temperature of 2 °C, for 0.5 h with stirring (1200 rpm). After the reaction was complete, the catalyst was removed from the reaction mixture and two aliquots of 0.05 g were titrated against the acidified Ce(SO_4_)_2_ solution using ferroin as an indicator. The degradation activity is reported as mol_H_2_O_2__ kg_cat_^–1^ h^–1^.

### Catalyst Reusability in the Direct Synthesis and Degradation of H_2_O_2_

In order to determine catalyst reusability, a similar procedure to that outlined above for the direct synthesis of H_2_O_2_ is followed utilizing 0.05 g of catalyst. Following the initial test, the catalyst was recovered by filtration and dried (30 °C, 16 h, under vacuum); from the recovered catalyst sample, 0.01 g was used to conduct a standard H_2_O_2_ synthesis or degradation test.

### Methane Oxidation Using In Situ Synthesized H_2_O_2_

The oxidation of methane was carried out using a Parr stainless steel autoclave with a nominal volume of a 50 mL reactor and a maximum working pressure of 2000 psi. To evaluate catalytic activity, the autoclave was charged with catalyst (0.027 g) and solvent (10 g H_2_O, Fischer Scientific, HPLC grade). Subsequently, the reactor was purged with methane (100 psi) and charged with pure H_2_, N_2_, O_2_ and CH_4_ such that the total pressure equaled 435 psi. The gas phase composition was 0.8% H_2_/ 1.6% O_2_/ 76.7% CH_4_/ 20.8% N_2_ to ensure the mixture was outside of the explosive limits. The autoclave was then heated to the desired reaction temperature (50 °C); once at the set temperature, the reaction solution was stirred at 1500 rpm for 0.5 h. After the reaction was complete, the stirring was stopped and the temperature was reduced to 10 °C using ice water in order to minimize the loss of volatile products. Gaseous samples were analyzed via gas chromatography (Varian-GC, equipped with a CPSIL5CB column (50 m, 0.33 mm internal diameter) fitted with a methanizer and flame ionization detector). The reaction mixture was filtered to remove the catalyst and analyzed by ^1^H NMR, using a Bruker 500 MHz Ultrashield NMR spectrometer. All ^1^H NMR samples were analyzed against a calibrated insert containing tetramethylsilane in deuterated chloroform (99.9% D). The remaining H_2_O_2_ was determined by titration with acidified Ce(SO_4_)_2_.

### Characterization

Investigation of the bulk structure of the materials was carried out using powder XRD on a (θ–θ) PANalyticalX’pert Pro powder diffractometer using a Cu Kα radiation source operating at 40 keV and 40 mA. Standard analysis was performed using a 40 min scan between 2θ values of 10 and 80° with the samples supported on an amorphous silicon wafer. Diffraction patterns of phases were identified using the ICDD database.

XPS measurements were performed on a Thermo Scientific K-Alpha^+^ spectrometer using a monochromatic AlKα radiation source operating at 72 W (6 mA × 12 kV) which defines an analysis are of approximately 400 × 600 μm. An analyzer pass energy of 150 eV was used for survey scans and 50 eV for elemental regions, all samples were recorded using a dual ion-electron charge compensation operating with an argon background pressure of ∼10^–7^ mbar. Samples were mounted by pressing on to silicone-free double-sided adhesive tape. Reported binding energies were referenced to a Si(2p) binding energy of 102.6 eV common for aluminosilicate materials, this was chosen as a more stable reference due to the low carbon concentrations on some of the materials leading to a greater deal of uncertainty in the C(1s) peak position. Spectra were quantified using CasaXPS^39^ using a Shirley-type background and an electron escape depth dependence based on the TPP-2 M equation and Scofield sensitivity factors to obtain surface compositions (atom%) of the different samples.

FTIR spectroscopy was carried out with a Bruker Tensor 27 spectrometer fitted with a HgCdTe (MCT) detector and operated with OPUS software.

N_2_ isotherms were collected on a Micromeritics 3Flex. Samples (∼0.1 g) were degassed (250 °C, 6 h) prior to analysis. Analyses were carried out at 77 K with P0 measured continuously. Free space was measured post-analysis with He. Pore size analysis was carried out using Micromeritics 3Flex software, N_2_–cylindrical pores–oxide surface DFT model.

Total metal loading and metal leaching from the supported catalysts were quantified using microwave plasma atomic emission spectroscopy (MP-AES). Fresh catalysts were digested (25 mg of catalyst, 2.5 mL of aqua regia, 24 h) prior to analysis using an Agilent 4100 MP-AES, while post-reaction solutions were also analyzed after filtration of the solid material. Metal concentrations were determined by the response at two characteristic emission wavelengths for Au (242.8 and 267.6 nm) and Pd (340.5 and 363.5 nm), and the resultant concentrations were averaged. The concentration responses of Au and Pd were calibrated using commercial reference standards (Agilent); in all cases, *r*^2^ > 0.999.

TEM was performed on a JEOL JEM-2100 operating at 200 kV. Samples were prepared by dispersion in ethanol via sonication and deposited on 300 mesh copper grids coated with a holey carbon film.

Aberration-corrected scanning transmission electron microscopy was performed using a probe-corrected Thermo Fisher Scientific Spectra 200 Cold-FEG operating at 200 kV. The instrument was equipped with a HAADF detector, and the imaging was done at a probe current of 120 pA and convergence angle of 30 mrad. Samples were dry dispersed onto 300 mesh copper grids coated with a holey carbon film. Energy-dispersive X-ray (EDX) mapping was performed using a Super-X G2 detector at a dwell time of 25 μs. All images and EDX data were processed using Velox software.

## Data Availability

The data underlying this study are available in the published article and its Supporting Information.

## Supporting Information Available

Data relating to the characterization of AuPd/ZSM-5 catalysts via XRD, FTIR, XPS, and TEM; determination of metal loading based on the digestion of as-prepared materials and porosimetry analysis (PDF)
