# Supplementary material for: Selective Oxidation of Methane to Methanol via In Situ H2O2 Synthesis
Source: ACS Org Inorg Au. 2023 Apr 20;3(4):177–83. doi: 10.1021/acsorginorgau.3c00001 (PMC10401884; doi:10.1021/acsorginorgau.3c00001)
Supplement: Supplementary file 1 — gg3c00001_si_001.pdf [file gg3c00001_si_001.pdf]

Selective oxidation of methane to methanol via in-situ H_2O_2 synthesis.

Fenglou Ni[a] ƚ, Thomas Richards[a], Louise R. Smith[a] David J. Morgan[a], [b], Thomas E. Davies[a], Richard J. Lewis[a] ƚ* and Graham J. Hutchings[a]*.

[a]Max Planck–Cardiff Centre on the Fundamentals of Heterogeneous Catalysis FUNCAT, Cardiff Catalysis Institute, School of Chemistry, Cardiff University, Main Building, Park Place, Cardiff, CF10 3AT, UK.

[b]Harwell XPS, Research Complex at Harwell (RCaH) Didcot, OX11 0FA, UK

ƚ These authors contributed equally to this work.

*LewisR27@cardiff.ac.uk, Hutch@cardiff.ac.uk

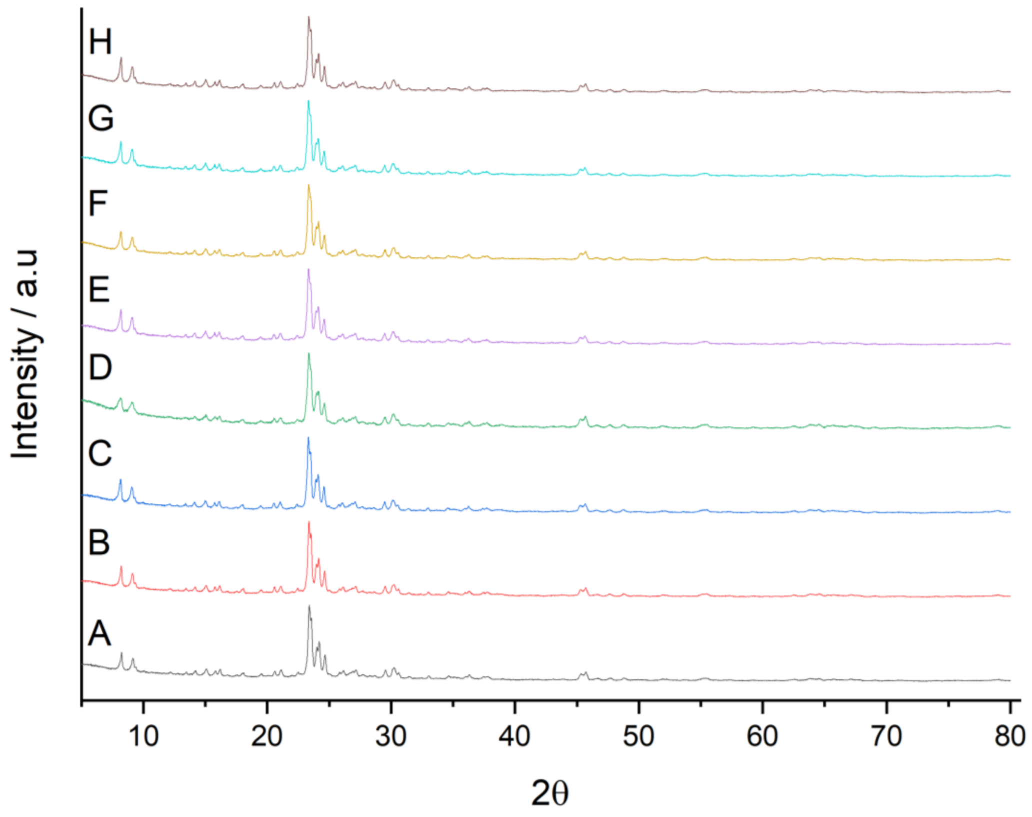

Figure S.1. X-ray diffractogram of 0.5%AuPd/ZSM-5 catalysts, prepared by a deposition precipitation methodology. **(A)** ZSM-5, **(B)** 0.5%Au/ZSM-5, **(C)** 0.475%Au-0.025%Pd/ZSM-5, **(D)** 0.375%Au-0.125%Pd/ZSM-5, **(E)** 0.25%Au-0.25%Pd/ZSM-5, **(F)** 0.125%Au-0.375%Pd/ZSM-5, **(G)** 0.025%Au-0.475%Pd/ZSM-5 and **(H)** 0.5%Pd/ZSM-5. **Note:** Precious metal immobilised catalysts were exposed to a reductive heat treatment (5%H_2/Ar, 400 °C, 3 h, 10 °Cmin^{-1})), in all cases the ZSM-5 support was calcined (6 h, 450 °C, 3 °Cmin^{-1}, flowing air) prior to metal deposition.

Supplementary Note 1.

Reflections characteristic of ZSM-5 can be observed at θ=7-9° and θ=23-25°.

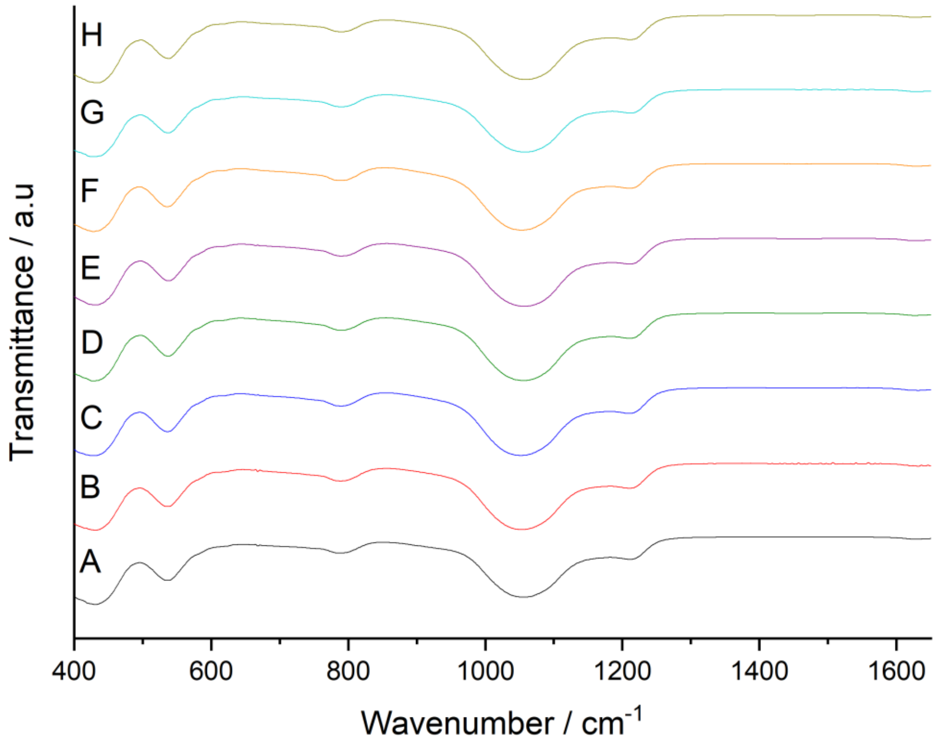

Figure S.2. FTIR spectra of 0.5%AuPd/ZSM-5 catalysts, prepared by a deposition precipitation methodology. **(A)** ZSM-5, **(B)** 0.5%Au/ZSM-5, **(C)** 0.475%Au-0.025%Pd/ZSM-5, **(D)** 0.375%Au-0.125%Pd/ZSM-5, **(E)** 0.25%Au-0.25%Pd/ZSM-5, **(F)** 0.125%Au-0.375%Pd/ZSM-5, **(G)** 0.025%Au-0.475%Pd/ZSM-5 and **(H)** 0.5%Pd/ZSM-5. **Note:** Precious metal immobilised catalysts were exposed to a reductive heat treatment (5%H_2/Ar, 400 °C, 3 h, 10 °Cmin^{-1}), in all cases the ZSM-5 support was calcined (6 h, 450 °C, 3 °Cmin^{-1}, flowing air).

Supplementary Note 2.

It is possible to observe three distinct bands in the FTIR spectra of the 0.5%AuPd/ZSM-5 catalysts, at approximately 800, 1050 and 1220 cm^{-1}, associated with the SiO_4 tetrahedron units. The adsorption band at approximately 1050 cm^{-1} is attributed to the internal asymmetric stretching vibrations of Si-O linkages and the adsorption band at approximately 1220 cm^{-1} is typically utilised to provide information on the structure of the zeolite and has been assigned to the 5- membered rings present within the framework structure of ZSM-5. [1]

Table S.1. Summary of porosity and surface area of key 0.5%AuPd/ZSM-5 catalysts.

**Catalysts**	**Surface area / m^2g^{-1}[a]**	**$V_{micropore}$ cm^3g^{-1}**
ZSM-5	369	0.12
0.25%Au-0.25%Pd/ZSM-5	353	0.15

[a] Surface area determined from nitrogen adsorption measurements using the BET equation. **Note:** Commercial ZSM-5 materials were exposed to an oxidative heat treatment (flowing air, 450 °C, 6 h, 3 °Cmin^{-1}), while metal immobilised catalysts were exposed to a reductive heat treatment (5%H_2/Ar, 400 °C, 3 h, 10 °Cmin^{-1}).

Table S.2. Nominal and actual total metal loading of 0.5%AuPd/ZSM-5 catalysts as determined by MP-AES.

Catalyst	Actual Au loading / wt.%	Actual Pd loading / wt.%
0.5%Au/ZSM-5	0.490	-
0.475%Au-0.025%Pd/ZSM-5	0.401	0.023
0.375%Au-0.125%Pd/ZSM-5	0.349	0.126
0.25%Au-0.25%Pd/ZSM-5	0.234	0.239
0.125%Au-0.375%Pd/ZSM-5	0.121	0.373
0.025%Au-0.475%Pd/ZSM-5	0.023	0.472
0.5%Pd/ZSM-5	-	0.49

Note: All catalysts were exposed to a reductive heat treatment (5%H_2/Ar, 400 °C, 3 h, 10 °Cmin^{-1}).

Table S.3. Catalytic reusability of 0.5%PdAu/ZSM-5 catalysts towards the direct synthesis and subsequent degradation of H_2O_2, as a function of Au: Pd ratio.

**Catalyst**	**Productivity / $mol_{H2O2}kg_{cat}^{-1}h^{-1}$**		**Degradation / $mol_{H2O2}kg_{cat}^{-1}h^{-1}$**	
	**Use 1**	**Use 2**	**Use 1**	**Use 2**
ZSM-5	0	0	12	11
0.5%Au/ZSM-5	2	2	20	25
0.475%Au-0.025%Pd/ZSM-5	3	2	35	50
0.375%Au-0.125%Pd/ZSM-5	7	5	58	74
0.25%Au-0.25%Pd/ZSM-5	9	5	68	85
0.125%Au-0.375%Pd/ZSM-5	10	7	104	116
0.025%Au-0.475%Pd/ZSM-5	12	9	112	123
0.5%Pd/ZSM-5	12	10	116	135

H_2O_2 direct synthesis reaction conditions: Catalyst (0.01 g), H_2O (2.9 g), MeOH (5.6 g), 5% H_2/CO_2 (420 psi), 25% O_2/CO_2 (160 psi), 0.5 h, 2° C, 1200 rpm. **H_2O_2 degradation reaction conditions:** Catalyst (0.01g), H_2O_2 (50 wt.% 0.68 g) H_2O (2.22 g), MeOH (5.6 g), 5% H_2 / CO_2 (420 psi), 0.5 h, 2 ^{O}C 1200 rpm.

Table S.4. Comparison of catalytic activity towards the oxidation of methane via the in-situ synthesis of H_2O_2.

Catalyst	Reactor	Catalyst mass / g	Temperature / °C	Time / h	Solvent (Additive)	Total Products / μmol							Ref
						CF_3COOH	CH_3OH	HCOOH	CH_3OOH	CO_2	Total Product	TOF[a] / h^{-1}	
1%Pd/AC + Fe-ZSM-5	Batch	0.05 +0.05	30	0.5	H_2O (15 mM H_2SO_4)	-	22	52	0	16	90	13.9	2
1%Pd/AC + Fe-mordenite	Batch	0.05 +0.05	30	0.5	H_2O (15 mM H_2SO_4)	-	14	57	0	22	93	9.3	2
1%Pd/AC + Fe-β	Batch	0.05 +0.05	30	0.5	H_2O (15 mM H_2SO_4)	-	15	144	1	131	291	38.6	2
1%Pd/AC + Fe-Y	Batch	0.05 +0.05	30	0.5	H_2O (15 mM H_2SO_4)	-	10	139	5	167	321	58.3	2
1%Pd/AC + Fe-ferrierite	Batch	0.05 +0.05	30	0.5	H_2O (15 mM H_2SO_4)	-	5	4	0	0	9	2.7	2
5%Pd/C + $Cu(CH_3COO_2)$ [Cu] = 0.05 mmol	Batch	0.021	80	5	TFA + TFAA	780	-	220	-	-	1000	3.3	3
5%Pd/C +NH_4VO_3 [V] = 0.1 mmol	Batch	0.005	80	5	TFA + TFAA	330	-	-	-	-	330	0.6	3
5%Pd/C + $Cu(CH_3COO)_2$ [Cu] = 0.1 mmol	Batch	0.021	80	10	TFA + TFAA	800	-	520	-	-	1320	1.2	4
5%Pd/C + $CuCl_2$ [Cu] = 0.1 mmol	Batch	0.021	80	10	TFA + TFAA	30	-	-	-	-	30	$2.7*10^{-2}$	4
5%Pd/C + NH_4VO_3 [V] = 0.1 mmol	Batch	0.021	80	10	TFA + TFAA	370	-	840	-	-	1210	1.1	4
5%Pd/C + V_2O_5 [V] = 0.1 mmol	Batch	0.021	80	10	TFA + TFAA	340	-	820	-	-	1160	1.0	4
5%Pd/C + $Mn(CH_3COO)_2$ [Mn] = 0.1 mmol	Batch	0.021	80	10	TFA + TFAA	30	-	-	-	-	30	$2.7*10^{-2}$	4
5%Pd/C + $FeCl_2$ [Fe] = 0.1 mmol	Batch	0.021	80	10	TFA + TFAA	150	-	-	-	-	150	0.1	4
5%Pd/C + $Fe(CH_3COO)_2$ [Fe] = 0.1 mmol	Batch	0.021	80	10	TFA + TFAA	320	-	250	-	-	570	0.5	4
5%Pd/C + $Co(CH_3COO)_2$ [Co] = 0.1 mmol	Batch	0.021	80	10	TFA + TFAA	50		60	-	-	110	0.1	4

5%Pd/C + $Ce(CH_3COO)_3$ [Ce] = 0.1 mmol	Batch	0.021	80	10	TFA + TFAA	40		20	-	-	60	$5.4*10^{-2}$	[4]
4.9 % Pd/c-s-HCPP + $FeSO_4$ [Fe] = 11.1 µmol	Batch	0.005	0	0.5	H_2O	-	7	16	4	6	33	4.9	[5]
1% Pd/AC + $FeSO_4$ [Fe] =11.1 µmol	Batch	0.05	0	0.5	H_2O	-	11	15	2	62	90	11.4	[5]
1% Pd/s-AC + $FeSO_4$ [Fe] =11.1 µmol	Batch	0.05	0	0.5	H_2O	-	9	18	2	88	117	14.8	[5]
1% Pd/ AC + $FeSO_4$ [Fe] =11.1 µmol	Batch	0.05	0	0.5	H_2SO_4	-	13	201	8	34	256	32.4	[5]
1% Pd/C + $FeSO_4$ [Fe] =11.1 µmol	Batch	0.05	20	0.5	H_2SO_4 +H_2O	-	11	218	7	124	360	45.6	[6]
1% Pd/C + $Fe_2(SO_4)_3$ [Fe] =11.1 µmol	Batch	0.05	20	0.5	H_2SO_4 +H_2O	-	10	216	8	132	366	46.3	[6]
1% Pd/C + $FeCl_2$ [Fe] =11.1 µmol	Batch	0.05	20	0.5	H_2SO_4 +H_2O	-	9	198	9	137	353	44.7	[6]
1% Pd/C + $FeCl_3$ [Fe] =11.1 µmol	Batch	0.05	20	0.5	H_2SO_4 +H_2O	-	10	205	10	83	308	39.0	[6]
1% Pd/C + $Fe(NO_3)_3$ [Fe] =11.1 µmol	Batch	0.05	20	0.5	H_2SO_4 +H_2O	-	8	218	9	139	374	47.3	[6]
1% Pd/C + $Fe(CH_3COO)_2$ [Fe] =11.1 µmol	Batch	0.05	20	0.5	H_2SO_4 +H_2O	-	10	218	10	131	369	46.7	[6]
3.24%Au-1.76%Pd@ZSM-5-C16	Batch	0.027	70	0.5	H_2O	-	64.1	3.6	-	-	67.7	15.2	[7]
AuPd@ZSM-5	Batch	0.027	70	0.5	H_2O	-	23.0	2.0	-	-		5.6[b]	[7]
AuPd/ZSM-5-C16	Batch	0.027	70	0.5	H_2O	-	7.1	1.5	-	-		1.9[b]	[7]
2.5% Au 2.5% Pd/TiO_2	Batch	$1.0*10^{-5}$ mol	50	0.5	H_2O	-	1.31	-	0.29	0.32	1.92	0.4	[8]
2.5% Au 2.5% Pd 2.5% Cu/TiO_2	Batch	$1.0*10^{-5}$ mol	50	0.5	H_2O	-	0.45	-	-	0.1	0.55	0.1	[8]
2.5% Au 2.5% Pd 1.0% Cu/TiO_2	Batch	$1.0*10^{-5}$ mol	50	0.5	H_2O	-	0.39	-	-	0.13	0.52	0.1	[8]
2.5% Au 2.5% Pd/TiO_2 + 2.5%Cu/TiO_2	Batch	$1.0*10^{-5}$ mol	50	0.5	H_2O	-	0.49	-	-	0.1	0.59	0.1	[8]
2.5%Pd-2.5%Au/activated carbon	Batch	0.03	50	0.5	H_2O	-	3.069	1.29	0.321	-	4.68	0.9	[9]
2.5%Pd-2.5%Au/CNTs	Batch	0.03	50	0.5	H_2O	-	4.17	1.272	0.261		5.703	1.1	[9]
0.5% Pd-0.5%Au/CNTs	Batch	0.03	50	0.5	H_2O	-	0.411	0.171	-		0.582	0.5	[10]
1% Pd-1%Au/CNTs	Batch	0.03	50	0.5	H_2O	-	0.621	0.231	-		0.852	0.4	[10]
0.14%Pd0.91%Cu/ZSM-5	Batch	0.03	120	0.5	H_2O	-		24.738		-		10.5[b]	[11]
0.19%Pd0.60%Fe/ZSM-5	Batch	0.03	120	0.5	H_2O	-		20.25				10.8[b]	[11]
0.18%Pd0.69%Co/ZSM-5	Batch	0.03	120	0.5	H_2O	-		1.869				0.9[b]	[11]

0.14%Pd0.59%Ni/ZSM-5	Batch	0.03	120	0.5	H_2O	-		1.859				1.1[b]	[11]
0.19%Pd0.47%Au/ZSM-5	Batch	0.03	120	0.5	H_2O	-		2.323				3.7[b]	[11]
2.5%Au,2.5%Pd/TiO_2	Batch	0.028	50	0.5	H_2O	-	1.31	-	0.29	0.32	1.92	0.4	[12]
2.5%Au,2.5%Pd/TiO_2	Batch	0.028	70	0.5	H_2O	-	0.81	-	0.1	0.11	1.02	0.2	[12]
2.5%Au/TiO_2+2.5%Pd/TiO_2	Batch	0.028	50	0.5	H_2O	-	0.12	-	-	0.54	0.66	0.1	[12]
2.5%Au-2.5%Pd/TiO_2	Batch	0.028	50	0.5	NADH/ H_2O	-	4.48	-	-	0.54	5.02	1.0	[12]
ZSM-5	Batch	0.028	50	0.5	H_2O	-	0	-	-	-	-	0	This Work
0.5%Au/ZSM-5	Batch	0.028	50	0.5	H_2O	-	0	-	-	-	-	0	This Work
0.475%Au-0.025%Pd/ZSM-5	Batch	0.028	50	0.5	H_2O	-	0.143	-	-	-	-	0.39	This Work
0.375%Au-0.125%Pd/ZSM-5	Batch	0.028	50	0.5	H_2O	-	0.188	-	-	-	-	0.44	This Work
0.25%Au-0.25%Pd/ZSM-5	Batch	0.028	50	0.5	H_2O	-	0.290	-	-	-	-	0.57	This Work
0.125%Au-0.375%Pd/ZSM-5	Batch	0.028	50	0.5	H_2O	-	0.205	-	-	-	-	0.35	This Work
0.025%Au-0.475%Pd/ZSM-5	Batch	0.028	50	0.5	H_2O	-	0.056	-	-	-	-	0.09	This Work
0.5%Pd/ZSM-5	Batch	0.028	50	0.5	H_2O	-	0	-	-	-	-	0	This Work

[a]Turnover frequency (TOF) = $mol_{total\ products}\ mol_{metal}^{-1}\ h^{-1}$

[b]Turnover frequency (TOF) = $mol_{oxygenates}\ mol_{metal}^{-1}\ h^{-1}$

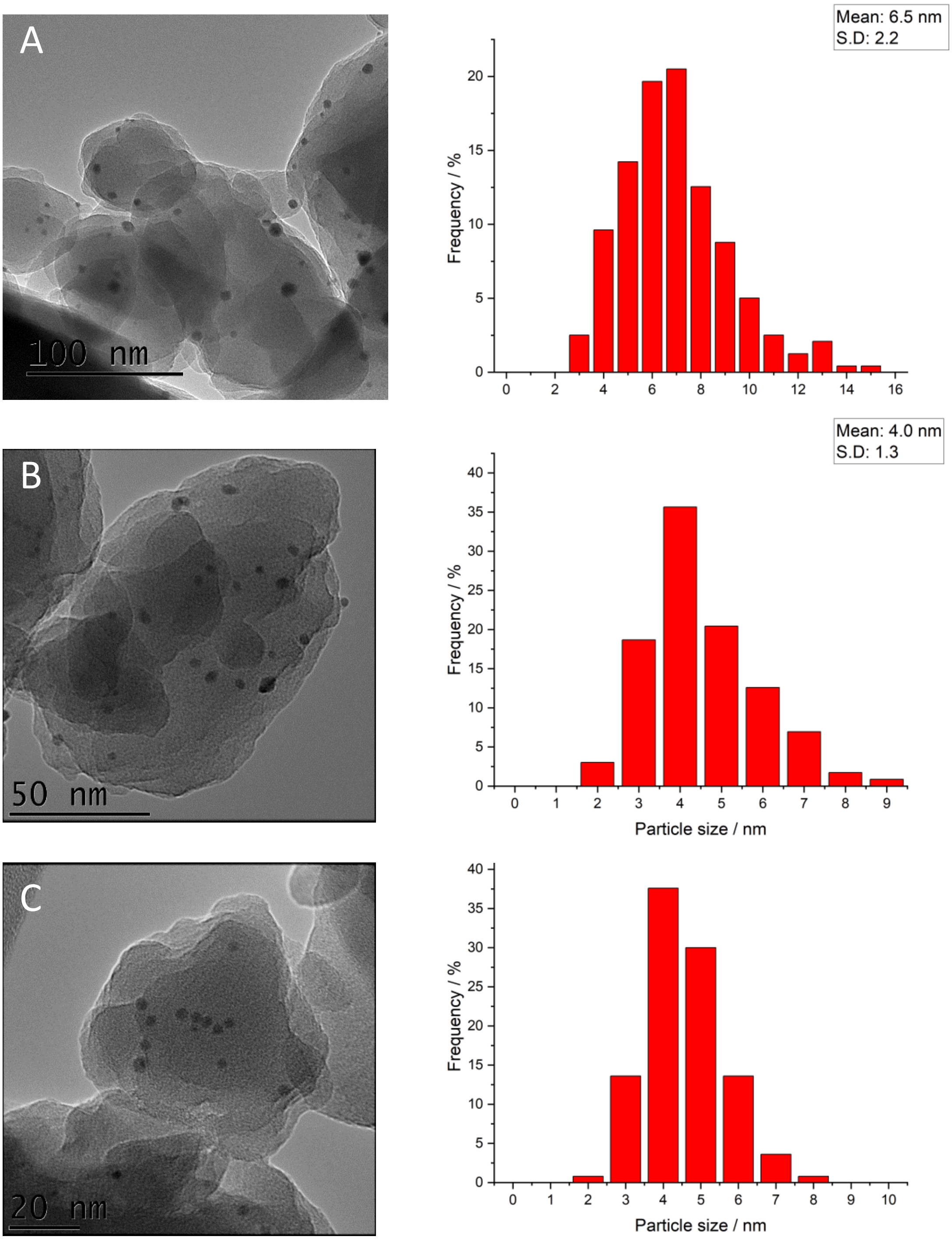

Figure S.3. Representative bright field transmission electron micrographs and corresponding particle size histograms of **(A)** 0.5%Au/ZSM-5 **(B)** 0.25%Au-0.25%Pd/ZSM-5 and **(C)** 0.5%Pd/ZSM-5. **Note:** All catalysts were exposed to a reductive heat treatment (5%H_2/Ar, 400 °C, 3 h, 10 °Cmin^{-1}).

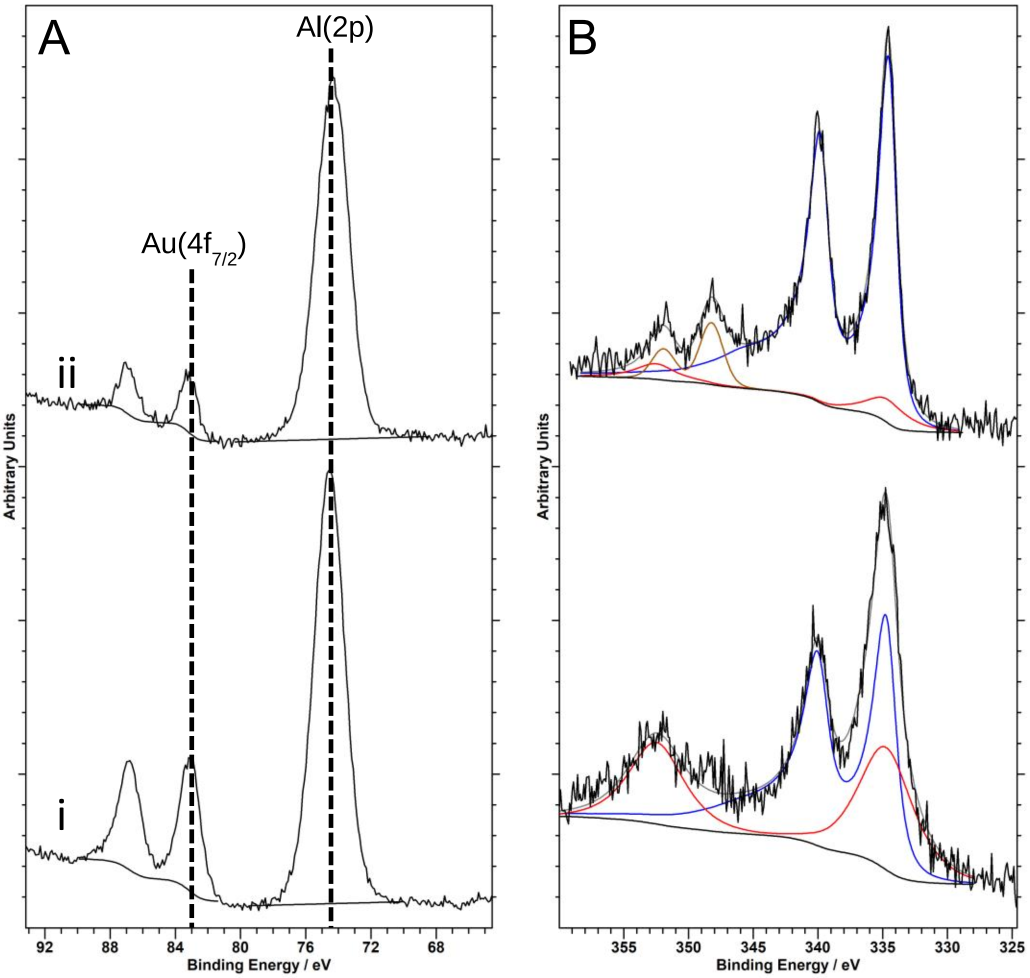

Figure S.4. Surface atomic compositions of **(i)** fresh and **(ii)** used 0.25%Au-0.25%Pd/ZSM-5 catalyst as determined by X-ray photoelectron spectroscopy using **(A)** Al(2p) and **(B)** Pd(3d)/Au(4d) regions. ***Key:*** Au^0 (*red*), Pd^0 (*blue*) and Ca^{2+} (*brown*).

References

1. Lewis, R. J.; Bara-Estaun, A.; Agarwal, N.; Freakley, S. J.; Morgan, D. J.; Hutchings, G. J. The Direct Synthesis of H_2O_2 and Selective Oxidation of Methane to Methanol Using HZSM-5 Supported AuPd Catalysts. *Catal. Lett.,* **2019**, *149* (11), 3066-3075. DOI: 10.1007/s10562-019-02876-7.

2. Kang, J.; Park, E. D. Selective Oxidation of Methane over Fe-Zeolites by In Situ Generated H_2O_2. *Catalysts* **2020**, *10* (3), 299. DOI: 10.3390/catal10030299

3. Park, E. D.; Hwang, Y.-S.; Lee, C. W.; Lee, J. S. Copper- and vanadium-catalyzed methane oxidation into oxygenates with in situ generated H_2O_2 over Pd/C. *Appl. Catal., A,* **2003**, *247* (2), 269-281. DOI: 10.1016/S0926-860X(03)00125-X.

4. Park, E. D.; Hwang, Y. S.; Lee, J. S. Direct conversion of methane into oxygenates by H_2O_2 generated in situ from dihydrogen and dioxygen. *Catal. Commun.,* **2001**, *2* (6), 187-190. DOI: DOI: 10.1016/S1566-7367(01)00030-9.

5. Kang, J.; Puthiaraj, P.; Ahn, W.; Park, E. D. Direct synthesis of oxygenates via partial oxidation of methane in the presence of O_2 and H_2 over a combination of Fe-ZSM-5 and Pd supported on an acid-functionalized porous polymer. *Appl. Catal., A,* **2020**, *602*, 117711. DOI: 10.1016/j.apcata.2020.117711.

6. Kang, J.; Park, E. D. Aqueous-Phase Selective Oxidation of Methane with Oxygen over Iron Salts and Pd/C in the Presence of Hydrogen. *ChemCatChem* **2019**, *11* (17), 4247-4251. DOI: DOI: 10.1002/cctc.201900919.

7. Jin, Z.; Wang, L.; Zuidema, E.; Mondal, K.; Zhang, M.; Zhang, J.; Wang, C.; Meng, X.; Yang, H.; Mesters, C; Xiao. F. Hydrophobic zeolite modification for in situ peroxide formation in methane oxidation to methanol. *Science* **2020**, *367* (6474), 193-197. DOI: doi:10.1126/science.aaw1108.

8. Ab Rahim, M. H.; Armstrong, R. D.; Hammond, C.; Dimitratos, N.; Freakley, S. J.; Forde, M. M.; Morgan, D. J.; Lalev, G.; Jenkins, R. L.; Lopez-Sanchez, J. A.; Taylor S.H; Hutchings G.H. Low-temperature selective oxidation of methane to methanol using titania supported gold-palladium copper catalysts. *Catal. Sci. Technol.,* **2016**, *6* (10), 3410-3418. DOI: 10.1039/C5CY01586C.

9. He, Y.; Luan, C.; Fang, Y.; Feng, X.; Peng, X.; Yang, G.; Tsubaki, N. Low-temperature direct conversion of methane to methanol over carbon materials supported Pd-Au nanoparticles. *Catal. Today,* **2020**, *339*, 48-53. DOI: 10.1016/j.cattod.2019.02.043.

10. He, Y.; Liang, J.; Imai, Y.; Ueda, K.; Li, H.; Guo, X.; Yang, G.; Yoneyama, Y.; Tsubaki, N. Highly selective synthesis of methanol from methane over carbon materials supported Pd-Au nanoparticles under mild conditions. *Catal. Today* **2020**, *352*, 104-110. DOI: 10.1016/j.cattod.2019.10.017.

11. Wu, B.; Lin, T.; Huang, M.; Li, S.; Li, J.; Yu, X.; Yang, R.; Sun, F.; Jiang, Z.; Sun, Y.; Zhong, L. Tandem Catalysis for Selective Oxidation of Methane to Oxygenates Using Oxygen over PdCu/Zeolite. *Angew. Chem. Int. Ed.,* **2022**, *61* (24), DOI: 10.1002/anie.202204116.
12. Ab Rahim, M. H.; Forde, M. M.; Jenkins, R. L.; Hammond, C.; He, Q.; Dimitratos, N.; Lopez-Sanchez, J. A.; Carley, A. F.; Taylor, S. H.; Willock, D. J.; Murphy, D.M.; Kiely, C.J.; Hutchings, G.J. Oxidation of Methane to Methanol with Hydrogen Peroxide Using Supported Gold–Palladium Alloy Nanoparticles. *Angew. Chem. Int. Ed.,* **2013**, *52* (4), 1280-1284. DOI: 10.1002/anie.201207717.
